# Variants identified by next-generation sequencing cause endoplasmic reticulum stress in Rhodopsin-associated retinitis pigmentosa

**DOI:** 10.1186/s12886-021-02110-2

**Published:** 2021-10-19

**Authors:** Yue Wang, Xi Chen, Xiang Gao, Andi Zhao, Chen Zhao, Xuejuan Chen

**Affiliations:** 1 Department of Ophthalmology, The First Affiliated Hospital With Nanjing Medical University, 300 Guangzhou Road, Nanjing, 210029 China; 2 Nanjing Medical University, Nanjing, 211166 China; 3 Department of Ophthalmology, Jiaozuo Health College, Henan, 454100 China; 4 Eye Institute, Eye & ENT Hospital, Shanghai Medical College, Fudan University, Shanghai, 200031 China; 5 NHC Key Laboratory of Myopia (Fudan University), Key Laboratory of Myopia, Chinese Academy of Medical Sciences, and Shanghai Key Laboratory of Visual Impairment and Restoration (Fudan University), Shanghai, 200031 China

**Keywords:** Autosomal dominant retinitis pigmentosa, Rhodopsin, Next-generation sequencing, Mutation, Endoplasmic reticulum stress

## Abstract

**Background:**

Rhodopsin (RHO) is the most well-known genetic cause of autosomal dominant retinitis pigmentosa (adRP). This study aimed to investigate the genetic cause of a large Chinese adRP family and assess the pathogenicity of the detected *RHO* mutant.

**Methods:**

Routine ocular examinations were conducted on all participants. Next-generation sequencing with targeted capture was performed to screen mutations in 179 genes associated with hereditary retinal diseases and 10 candidate genes. Variants detected by NGS were validated by Sanger sequencing and evaluated for pathogenicity. Fragments of mutant and wild-type *RHO* were cloned into the pEGFP-N1 vector and were transfected into different cell lines to observe the cellular localization of the Rhodopsin-GFP fusion protein and evaluate the expression of endoplasmic reticulum (ER) stress markers. RT-PCR analysis was used to detect transfected the splicing of X box-binding protein 1 (*XBP1*) mRNA, which is a critical factor affecting ER stress.

**Results:**

Genetic analysis identified a heterozygous missense variant, *RHO,* c.284 T > C (p.L95P) in this adRP family. Another *RHO* variant (p.P53R) that we reported previously was also included in further functional assessment. Both misfolded mutant proteins accumulated in the ER in a manner similar to that noted for the classic mutant P23H. Spliced *XBP1* was observed in cells transfected with mutants, indicating an increase in ER stress.

**Conclusions:**

Although the p.L95P variant is not a novel change, it was the first variant to be functionally evaluated and reported in Chinese RP patients. The results in our study provide significant evidence to classify the p.L95P mutation as a class II mutation.

**Supplementary Information:**

The online version contains supplementary material available at 10.1186/s12886-021-02110-2.

## Background

Retinitis pigmentosa (RP, OMIM 26,800) represents a highly genetically and clinically heterogeneous form of hereditary retinal diseases (HRDs) that is characterized by progressive photoreceptor degeneration with a prevalence of approximately 1 in 3500—4000 [1, 2]. Individuals affected with RP clinically present typically impaired dark adaptation and the development of night blindness followed by progressive visual field loss [3]. In approximately 30–40% of RP patients, the condition is are inherited as an autosomal-dominant (AD) trait. The disease is inherited in an autosomal-recessive (AR) manner in 50–60% of patients, and the condition is X-linked in 5–15% of patients [4, 5]. To date, variants of at least 280 genes have been identified as responsible for causing different forms of HRDs [1, 6, 7]. Among these, the mutations in rhodopsin (*RHO*) are the most common pathogenetic cause of adRP, accounting for 20–30% of all cases [8].

Rhodopsin is a light-detecting G-protein-coupled receptor that plays a pivotal role in phototransduction and consists of a typical seven-transmembrane domain, an extracellular N-terminal tail and a cytoplasmic C-terminal tail [9]. Mutations in *RHO* can both be inherited in an AD or AR manner with a classical form of RP, which is caused by the degeneration of rods, followed by the degeneration of cones [10]. Rhodopsin is synthesized and matured in the inner segment (IS) of rod photoreceptors and transported to the membranous disc of its outer segment (OS). Rhodopsin consists of the rod-specific opsin and the vitamin A derived chromophore 11-cis-retinal, which is converted to all-trans-retinal to activate the phototransduction cascade by absorption of a photon [11]. Moreover, abnormal trafficking or localization of rhodopsin leads to dysfunction and death of photoreceptors cells [12, 13].

As previously described [14], we accomplished a targeted NGS in 25 distinct Chinese probands with diverse categories of HRDs, including adRP and arRP, Stargardt disease, Usher syndrome, BBS syndrome, and Wagner syndrome. Among these, the known *RHO* missense mutation c.158C > G (p.P53R) was detected and confirmed to be co-segregated in an adRP family (named as HD09, Supplemental Figure 1A and B). Here, we identified the heterozygous missense mutation *RHO*, c.284 T > C (p.L95P) in another unrelated Chinese adRP family using the same approach. Although the two variants identified in our study are not novel changes, both of them are the first to be reported in Chinese patients [15, 16]. According to the first description, the p.L95P variant was not only observed in the proband but also carried by an ‘unaffected’ member in that Iranian family [16]. Moreover, evidence of co-segregation is lacking because other familial members refuse to participate, which seems to raise some questions on the pathogenicity of this variant [16]. Given that rhodopsin is a well-studied protein, functional evaluation plays a critical role in interpreting the significance of each variant in clinical practice and genetic therapy. Therefore, we further assessed the pathogenic effect of the two mutants in vitro, and both misfolded mutant proteins accumulated in the ER in a manner similar to the pattern observed for the classic mutant P23H. In addition, spliced *XBP1* was also observed in cells transfected with mutants. Generally, our study results provide significant evidence for the pathogenicity of the p.L95P variant base on the complete co-segregation in the second family and visible functional changes in vitro.

## Methods

### Clinical assessment of patients

**Fig. 1:**
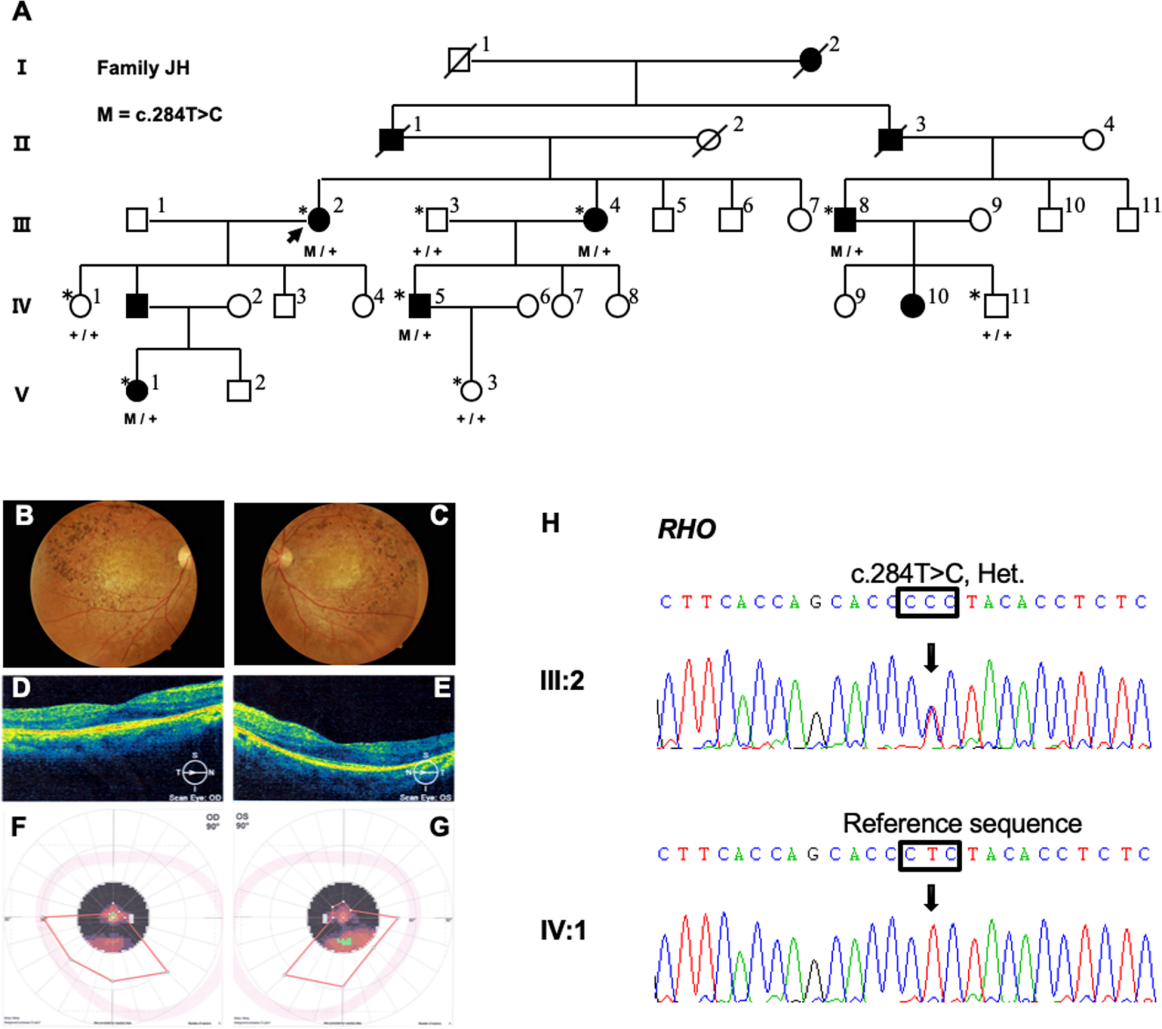
Pedigree, clinic, and genetic evaluations of the JH family. **A** Pedigree of family JH. Solid symbols, affected individuals; open symbols, unaffected individuals; arrow, proband; slash, deceased persons; + , wild-type; M, the heterozygous mutation c.284 T > C (p.L95P) in the *RHO* gene; *, the family members available for the present study; **B-C** Fundus photograph of two eyes; **D-E** Optical coherence tomography detected obviously thinned and disorganized outer structure of the retina in two eyes; **F-G** Constricted visual fields of two eyes. **H** Sanger sequencing showing heterozygous c.284 T > C mutation in III:2 (patient) and IV:1 (unaffected) respectively. Abbreviation: Het., heterozygous

A large, five-generation Chinese family (here named family JH) was recruited from The First Affiliated Hospital with Nanjing Medical University. Five patients and four unaffected relatives from family JH participated in our study (Fig. 1A). Complete ophthalmic examinations were performed on each participating individual, as previously described [14]. Written informed consent was obtained from the participants and the parents of each child for sample collection and genetic analysis. All procedures were reviewed and approved by the local institutional ethical review board under the Declaration of Helsinki principles.

### RDs189-array capture and NGS

As previously described [14], we designed an RDs189-array and constructed a 2.1 Megabase NimbleGen sequence capture microarray platform (Roche, Madison, WI, USA) to capture 4641 exons from 179 genes associated with HRDs and 10 candidate splice genes. Targeted NGS was performed on patients JH-III:2 and JH-III:8 as well as unaffected member JH-IV:1 to determine the genetic causation in identified RP relevant genes for family JH. Template preparation, hybridization with the RDs189-array, and NGS were performed in collaboration with the Beijing Genome Institute (BGI, Shenzhen, China).

### Sanger sequencing

Sanger sequencing was performed by amplifying the coding regions of the *RHO* gene with polymerase chain reaction (PCR) to validate the variations detected by NGS, the segregation analysis of interesting variations when possible, and the prevalence testing of variations in unrelated controls.

### In Silico Analyses

Pathogenicity prediction was performed by using three online mutational pathogenicity evaluation software: SIFT (http://sift.jcvi.org/); PolyPhen-2 (http://genetics.bwh.harvard.edu/pph2/); and Mutationtaster (http://www.mutationtaster.org/). Evolutionary conservation of the mutated residues was analysed with ENDscrit 3.0 Tool [17] by aligning the protein sequence of human rhodopsin with 9 other species. Crystal structural models of wild-type and mutant rhodopsin were constructed using the SWISS-MODEL and EMBL-EBI online servers. Predicted structures were displayed using Swiss-PdbViewer software [18].

### Plasmid construction

Wild-type rhodopsin cDNA was synthesized by BGI and cloned into the pEGFP-N1 (Clontech, Mountain View, CA) expression plasmid, further referred to as ‘WT’. Nhel and Agel restriction sites were introduced at the 5’—and 3’ -ends, respectively. The p.P23H, p.P53R, and p.L95P mutations were generated by site-specific mutagenesis using a two-step PCR overlap extension method (named P23H, P53R, and L95P) [19]. All the constructs were purified with the QIAGEN plasmid isolation kit (QIAGEN, USA) and confirmed by direct sequencing (Supplemental Figure 2).

### Cell culture and transfection

Human retinal pigment epithelial cell lines (ARPE19) and human embryo kidney 293 T (HEK293T) cell lines were purchased from the American Type Culture Collection (ATCC; Manassas, VA). ARPE19 cells were cultured in DMEM/F12 media (Gibco, Invitrogen, Grand Island, NY, USA), whereas HEK293T cells were cultured in Dulbecco's Modified Eagle's Medium (Gibico, Invitrogen, Grand Island, NY, USA). Both cells lines were supplemented with 10% foetal bovine serum (Gibco, Invitrogen, USA), 100 U/ml penicillin, and 100 mg/ml streptomycin (Hyclone, Thermo, USA) at 37 °C under 5% CO2. Cells were cultured to 60–70% confluence and transiently transfected with the plasmids (WT, P23H, P53R, L95P, and empty vector) using the Lipofectamine 3000 (Invitrogen, Carlsbad, CA, USA) according to the manufacturer’s protocols. Transfected cells were visualized based on green fluorescent protein (GFP) fluorescence.

### Immunocytochemistry

ARPE19 cells were cultured on coverslips of confocal dishes and transfected with the pEGFP-N1 vector carrying WT or mutant *RHO*. Twenty-four hours post-transfection, cells were washed and stained with ER-Tracker (Beyotime, Shanghai, China) according to the protocols. Cells were washed with 1 × PBS 3 times to clear the dyes. After staining with Hoechst 33,258 (Beyotime, Shanghai, China) at 37 °C for 20–30 min, cells were observed by fluorescence confocal microscopy (Carl Zeiss, Germany).

### Protein isolation and Western blot

At 48 h after transfection, HEK293T cells were harvested, and proteins were extracted from cell lysates. An equal amount of each protein sample was resolved by 10% sodium dodecyl sulfate polyacrylamide gel electrophoresis (SDS-PAGE) and transferred electrophoretically onto a polyvinylidene difluoride (PVDF) membrane. The membranes were blocked for 30 min and then incubated overnight with each objective protein. Antibodies against RHO (1:1000), XBP1(1:1000), and GAPDH (1:5000) were purchased from Abcam (Abcam, Hong Kong).

### RT-PCR analysis of XBP1 splicing

Twenty-four hours post-transfection, total RNA was extracted from cultured HEK293T cells using TRIzol RNA Isolation Reagent (Thermo, USA) according to the manufacturer’s protocols. Reverse-transcriptase polymerase chain reaction (RT-PCR) was performed to obtain the amplified fragments of the human *XBP1* gene using the specific primers reported in a previous study [20]: Forward, 5’-TTACGAGAGAAAACTCATGGC-3’; Reverse, 5’- GGGTCCAAGTTGTCCAGAATGC-3’. A 289-bp and a 263-bp amplicon were generated from unspliced *XBP1* and spliced *XBP1*, respectively. PCR products were further resolved on a 3.5% agarose/1 × TAE gel.

## Results

### Clinical assessments of patients in two families

In family JH, all patients began suffering night blindness around the age of 10 to 15 years, exhibiting other accompanying ocular symptoms such as reduced visual acuity and peripheral visual fields deteriorating with age. The proband (individual III: 2) was a 67-year-old female who had a history of night blindness for fifty-seven years. The latest ocular examination showed poor vision (only FC in both eyes), narrow visual fields (Table 1), and typical fundus features including diffuse retinal pigment deposition with posterior pole involvement, attenuation of the retinal vessels, and pale optic discs (Fig. 1B-C). Thinned and disorganized outer structures of the retina were detected in two eyes (Fig. 1D-E). In addition, she had constricted visual fields, was diagnosed with glaucoma for two years and underwent glaucoma surgery (Fig. 1F-G). Compared with family JH, patients in family HD09 showed earlier onset, and all of them began suffering night blindness in their early childhood. The proband (individual IV:4) was a 35-year-old female with a history of night blindness since five years of age and had corrected visions of 0.4 in both eyes. She exhibited a characteristic RP fundus appearance, including pale optic discs, attenuated retinal arterioles, and considerable bone spicule-like pigmentation (Supplemental Figure 1C). Her affected daughter (individual V:3) suffered night blindness at the age of three. Although no significant visual field changes were detected, a few bone spicule-like pigmentations could be noticed in the periphery. Individuals III:2, III:3, and III:5 showed noticeable visual field changes before 20 years of age and suffered successive acute angle-closed glaucoma and complicated cataracts at approximately 40 years of age. Among these individuals, III:5 had lost sight for several years. Detailed clinical information for affected members of each family is summarized in Table 1.

**Table 1:** Clinical features for affected members of two adRP families

Family ID	Patient number	Age(year)^a^/Sex	Onset age (year)^b^	VA		Cataract	Visual field	Glaucoma
				O.D	O.S			
Family JH	III-2	67/F	10	FC	FC	YES	Unable to cooperate	YES
	III-8	58/M	15	0.2	0.2	YES	Constriction	YES
	III-4	65/F	12	0.12	0.1	YES	Constriction	NO
	IV-6	45/M	10	0.4	0.4	NO	Constriction	NO
	V-1	22/F	12	0.6	0.6	NO	N/A	NO
Family HD09	III-2	58/F	3	0.1	0.1	YES	N/A	YES
	III-3	56/M	3	FC	0.1	YES	N/A	YES
	III-5	51/M	4	NLP	NLP	YES	N/A	YES
	IV-4	35/F	5	0.4	0.4	NO	Constriction	NO
	IV-5	33/M	4	0.2	0.1	NO	N/A	NO
	IV-10	34/F	3	0.25	0.2	NO	N/A	NO
	IV-12	29/M	4	0.25	0.5	NO	N/A	NO
	IV-14	28/F	5	0.4	0.5	NO	N/A	NO
	V-3	7/F	3	0.6	0.6	NO	N/A	NO

^a^Age of recruitment and examination; ^b^Onset age of night blindness; Abbreviations: *VA* visual acuity, *O.D.* right eye, *O.S.* left eye, *FC* finger count, *NLP* no light perception, *N/A* not available

### Genetic findings and Bioinformatics analysis

The average call rates for targeted bases were greater than 99.84%. The average mean depth for the targeted regions among all samples was 76.00 ± 11.83-fold, and an average of 99.65%, 99.16%, and 97.48% of targeted bases was covered at 4 × , 10 × , and 20 × fold, respectively. A total of 7,239 were detected initially in family JH; furthermore, only one rare variant c.284 T > C [p.L95P] in the *RHO* gene remained after bioinformatics analysis and filtering as previously described [18]. Based on Sanger sequencing, this heterozygous variant was confirmed to co-segregate entirely with the disease in family JH (Fig. 1A and 1H) and absent in another 100 unrelated ethnically matched controls. The analysis of the variant obtained scores of 0 (damaging), 1 (probably damaging) and disease causin using three bioinformatics approaches (SIFT, PolyPhen-2 and MutationTaster). The details of targeted NGS and bioinformatics filtering for variant *RHO*, c.158C > G (p.P53R) in family HD09 had been previously reported(19).

**Fig. 2:**
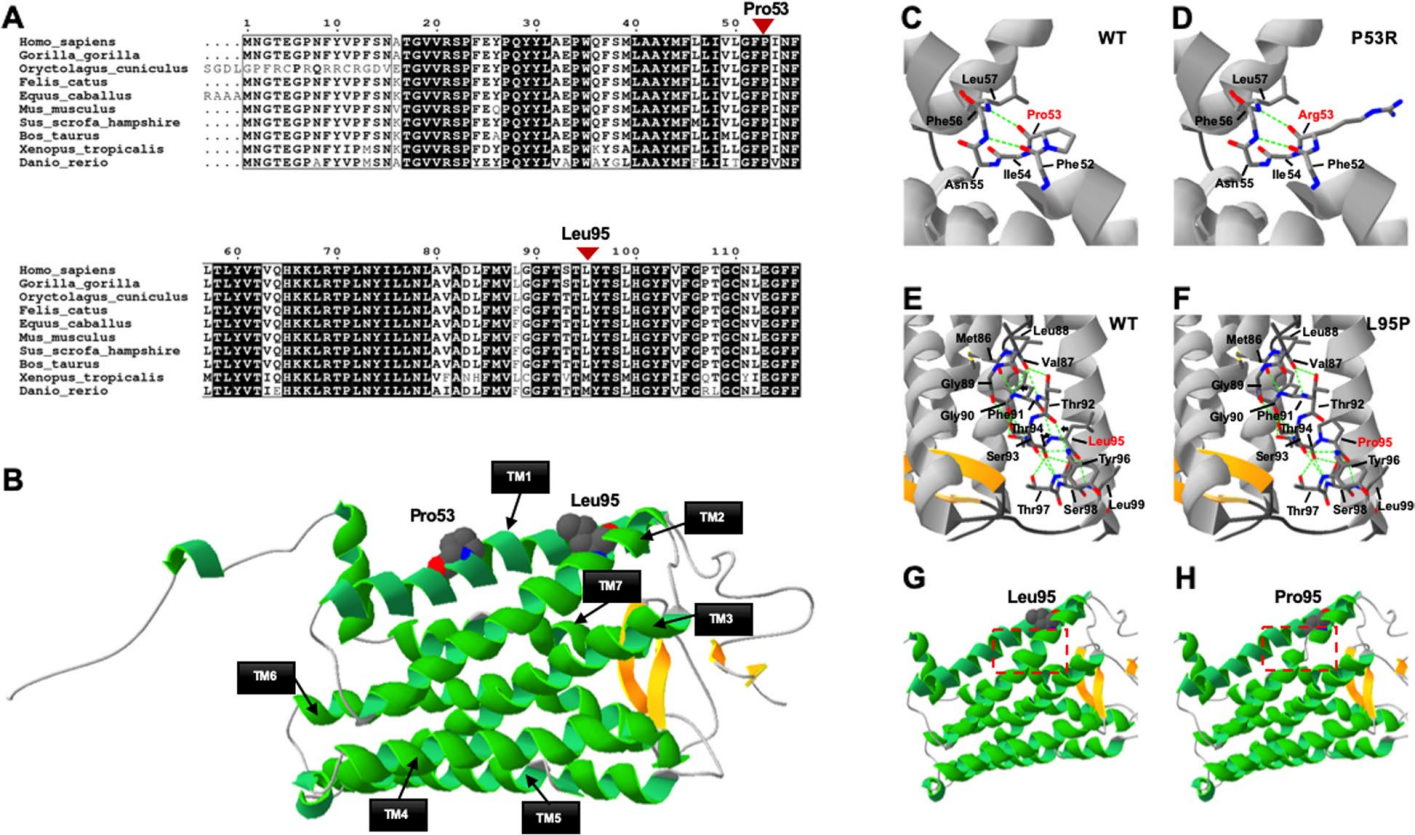
Schematic structural of rhodopsin and predicted crystal structural models of the wild-type and mutants. **A** Conservation analysis of residues Pro53 and Leu95 across ten species. **B** Rhodopsin is a light-detecting G-protein-coupled receptor, consisting of a typical seven-transmembrane domain, an extracellular N-terminal tail and a cytoplasmic C-terminal tail. **C-D** Predicted crystal structural models of the wild-type and mutant p.P53R. **E–F** Predicted crystal structural models of the wild-type and mutant p.L95P. In comparison with WT, the structure of the L95P mutant protein showed that the hydrogen-bonds between Val87 and Phe91, Phe91 and Leu95, Thr92 and Tyr96 were destroyed (arrowed). **G-H** The α-helical structure where Leu95 located (boxed) is prematurely interrupted due to mutation to proline according to the structure prediction result

Comparative and structural analyses were conducted to predict the potential pathogenic effect of the two missense variants (Fig. 2). Both variants were highly conserved using multiple orthologous sequence alignment (Fig. 2A). Interestingly, the α-helical structure where Leu95 is located prematurely interrupted due to mutation to proline according to the structure prediction result (Fig. 2B-H).

### Subcellular localization of Rhodopsin Mutants

**Fig. 3:**
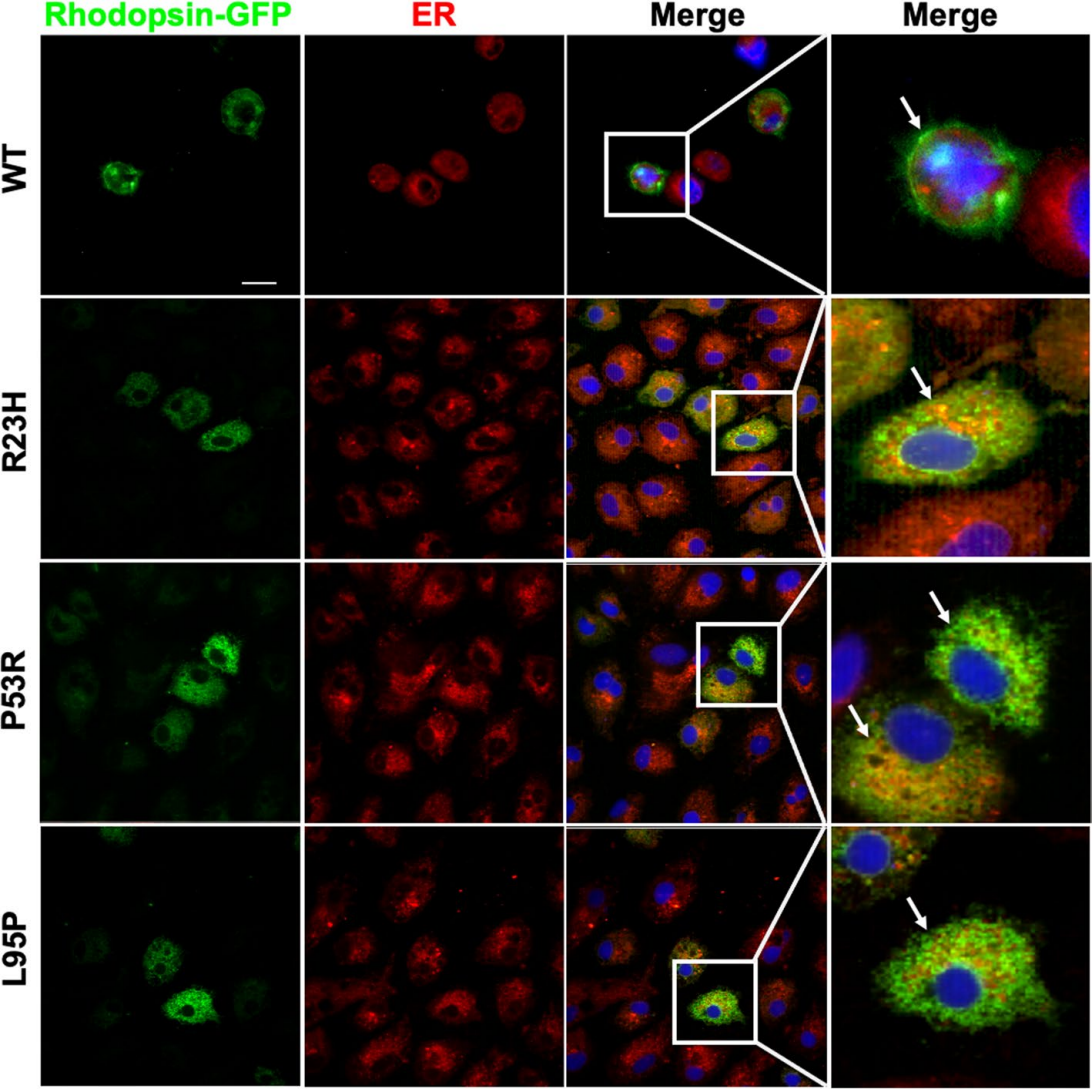
Co-localization of rhodopsin with the ER marker. Immunofluorescent localization of GFP-fused Rhodopsin (green) and ER (red) in WT and mutant rhodopsin expressing cells. Nuclei were stained with Hoechst (blue). WT-rhodopsin expressing cells displayed plasma membrane (white arrow) and cytoplasmic staining patterns. In contrast, all three mutant Rhodopsin expressing cells (P23H, P53R and L95P) showed punctate co-localization patterns with the ER-Tracker Red (merge: yellow). Scale bar = 20 μm

Previous studies documented that misfolded and accumulated mutant rhodopsin within the ER leads to a non-functional rhodopsin chromophore with 11 cis-retinal [21] and cellular apoptosis [22]. To demonstrate the functional consequences of the two detected mutants in the *RHO* gene, ARPE 19 cells were transfected with WT, P23H, P53R, and L95P GFP-tagged constructs and subject to immunocytochemistry assays 24 h later. Fluorescence microscopy indicated that both P53R and L95P mutants were mainly localized to the ER with a pattern similar to that observed for P23H, whereas wild-type rhodopsin was trafficked predominantly to the cell membrane (Fig. 3), These results indicate abnormal protein transportation based on the two heterozygous *RHO* mutations we found in this study.

### Detection of ER stress induced by Rhodopsin Mutants

**Fig. 4:**
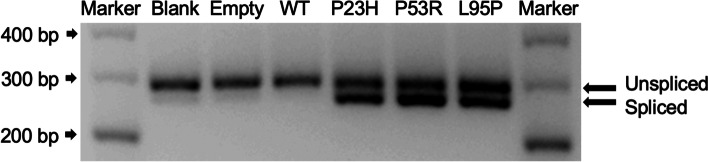
Detection of *XBP1* splicing. Agarose gel electrophoretic image of the amplified fragments of the human *XBP1* gene obtained from the RT-PCR. Both unspliced *XBP1* (289 bp) and spliced *XBP1* (263 bp) were detected following overexpression of mutant rhodopsin transfection in HEK293T cells

A previous study reported activation of the IRE1-dependent ER stress pathway by analysing *XBP1* mRNA splicing as a marker of IRE1 activity [23]. IRE1 is one of the major ER-resident transmembrane proteins, and induction of the unfolded protein response (UPR) causes IRE1 activation [24] that catalyses *XBP1* mRNA splicing by excising a 26-nucleotide intron. *XBP1* mRNA splicing leads to a shift in the coding reading frame and the generation of spliced *XBP1* (XBP1s), a transcription factor that controls genes involved in protein folding [24]. Therefore, we further conducted PCR and Western blot analysis to determine whether *XBP1* was spliced following overexpression of mutant rhodopsin transfection in HEK293T cells. The mRNA level indicating that the IRE1-dependent ER stress pathway was activated, but is not clear in protein (Fig. 4 and Supplemental Figure 3).

## Discussion

In the present study, a rare heterozygous variant c.284 T > C (p.L95P) was detected by targeted NGS and confirmed to exhibit co-segregation entirely in a large Chinese adRP family. This variant is absent in another 100 unrelated ethnically matched controls and highly conserved across several species. The mutation was also predicted to be damaging based on three bioinformatics approaches (SIFT, PolyPhen-2, and MutationTaster). Moreover, it is not a novel mutation and has been reported in an Iranian family recently [16]. However, in its first description, the co-segregation of the p.L95P variant with the phenotype was imperfect. Specifically, only two members were included from that family, and the ‘unaffected’ member also carried this variant, which seems to raise some questions on the pathogenicity of this variant [16]. Our results provided significant evidence for the pathogenicity of this variant, according to the complete co-segregation in the second family. Furthermore, we assessed the pathogenic effect of this mutant in vitro, and the misfolded mutant protein accumulated in the ER, exhibiting a pattern similar to that noted for the classic mutants P23H and P53R. In addition, spliced *XBP1* was also observed in cells transfected with mutants. These data further confirm and elaborate on the pathogenicity and functional mechanism of the p.L95P mutation.

As the first genetic cause of RP, causative mutations in the *RHO* gene were identified in 1990 [25, 26]. To date, approximately 20–30% of adRP cases are associated with mutations in the *RHO* gene [8]. Thus, it is important for investigators to extend the spectrum of *RHO* mutations, probe into potential mechanisms underlying Rhodopsin-associated RP and explore innovative treatments. Athanasiou et al. classified *RHO* gene mutation into 8 groups based on their biochemical and cellular properties [27]. Class I and II involve most mutations*.* Mutations in the former class are reported to disturb trafficking to the OS by causing changes to the C-terminal OS trafficking motif, whereas the latter, which could occur in the intradiscal, transmembrane or cytoplasmic domains of rhodopsin, causes protein misfolding and is retained completely or partly in the ER [22, 27]. Residue 95 is located in the second transmembrane (TM2) domain of the rhodopsin protein structure, so mutations in its neighbours (T94I, T92I, and T97I) are ascribed to different classifications and cause different clinical phenotypes [26]. Therefore, it is not cautioned to speculate its pathogenic mechanism according to its location; further in vitro experiments are needed.

According to the secondary structure prediction results, the third helical of rhodopsin consists of 9 residues, Phe91-Leu99 (FTTTLYTSL), via the second helical (from residue Pro71 to Gly89) to form the TM2 domain. Compared with WT, the structure of the L95P mutant protein reveals that the hydrogen-bonds between Val87 and Phe91, Phe91 and Leu95, as well as Thr92 and Tyr96 were destroyed due to the mutation that changed Leu95 to Pro95 (Fig. 2E–H). Proline disrupts the alpha helix because it cannot form a hydrogen bond since one less hydrogen atom is present is present in its imino group and unrotated Cα-N bond. These features potentially explain the resulting structure in the TM2 domain in rhodopsin based on the mutant L95P.

The *RHO*, c.68C > A (p.P23H) mutation was the first mutation reported and the most representative of a class II mutation [20, 26]. In addition, the p.P53R mutant has been classified as a class II mutation based on evidence from overexpression experiments [28]. Therefore, both mutants were included as the positive references. ARPE19 cells were transfected with the pEGFP-N1 vector carrying WT or three rhodopsin mutants to compare the localization of the Rhodopsin-GFP fusion protein in cells. Morphological findings revealed that consistent with P23H and P53R, L95P formed abnormal rhodopsin that was retained in the ER rather than being transported to the plasma membrane. Hence, we hypothesize that the present missense mutation from leucine to proline in residual 95 is a class II *RHO* mutations. Davies et al. identified four rhodopsin variants (F45L, P53R, R69H, and M39R) and reported that F45L and R69H variants behave like wild-type, whereas P53R and M39R were retained in the ER with significantly reduced functionality [28]. Our findings corroborated Davies’s investigation about *RHO* p.P53R, and this study is the first to classify the *RHO* p.L95P mutation as a class II mutation based on its functional analysis.

The accumulation of misfolded rhodopsin in the ER has been shown to cause ER stress and ultimately result in the death of photoreceptor cells in RP cases. Cells react to ER stress by activating the UPR, which is initiated by three proteins sensors, activating transcription factor 6 (ATF6), inositol­requiring enzyme 1α (IRE1α), and protein kinase RNA-like endoplasmic reticulum kinase (PERK), that work to restore ER protein folding ability [29]. In general, these sensors bind to Immunoglobulin-binding protein (BiP/GRP78) to maintain an inactive status. Once misfolded proteins aggregate in the ER, BiP dissociates from the sensors and combines with unfolded proteins, which activate the sensors. The mRNA encoding X­box binding protein 1 (*XBP1*) undergoes removal of 26 nucleotides by activated IRE1a, which changes the reading frame and causes a spliced variant that encodes a functional form of the XBP1 protein, XBP1s, that attenuates the burden of ER by inducing BiP/GRP78 expression [30, 31]. Therefore, the expression of XBP1s is a reasonable marker of ER stress. In this study, we observed abnormal *XBP1* spliced mRNA in extracts of HEK293T cells transfected with P23H, P53R, and L95P compared with wild-type, blank-control, and negative-control cells, indicating that IRE1α signaling pathways were activated by the expression of mutant Rhodopsin as mentioned above. These findings further confirmed that the retention of misfolded mutant Rhodopsin in the ER caused ER stress and activated the UPR.

## Conclusions

In summary, we identified a heterozygous missense mutation, *RHO*, c.284 T > C (p.L95P) in a larger Chinese adRP family using targeted NGS approach. Although the p.L95P variant is not a novel change, it was the first to be functionally evaluated and reported in Chinese patients. The results in our study supplied significant evidence to classify the p.L95P as a class II mutation.

## Supplementary Information

**Additional file 1.**

**Additional file 2.**

**Additional file 3.**

**Additional file 4.**

## Data Availability

The datasets used and/or analyzed during the current study are available from the corresponding author on reasonable request.

## Abbreviations

*RHO*: Rhodopsin
adRP: Autosomal dominant retinitis pigmentosa
ER: Endoplasmic reticulum
HRDs: Hereditary retinal diseases
XBP1: X box-binding protein 1
AR: Autosomal-recessive
NGS: Next-generation sequencing
PCR: Polymerase chain reaction
GFP: Green fluorescent protein
UPR: Unfolded protein response
ATF6: Activating transcription factor 6
IRE1α: Inositol­requiring enzyme 1α
PERK: Protein kinase RNA-like endoplasmic reticulum kinase
