# Supplementary material for: Variants identified by next-generation sequencing cause endoplasmic reticulum stress in Rhodopsin-associated retinitis pigmentosa
Source: BMC Ophthalmol. 2021 Oct 19;21:371. doi: 10.1186/s12886-021-02110-2 (PMC8525045; doi:10.1186/s12886-021-02110-2)
Supplement: Supplementary file 1 — Additional file 1. [file 12886_2021_2110_MOESM1_ESM.pdf]

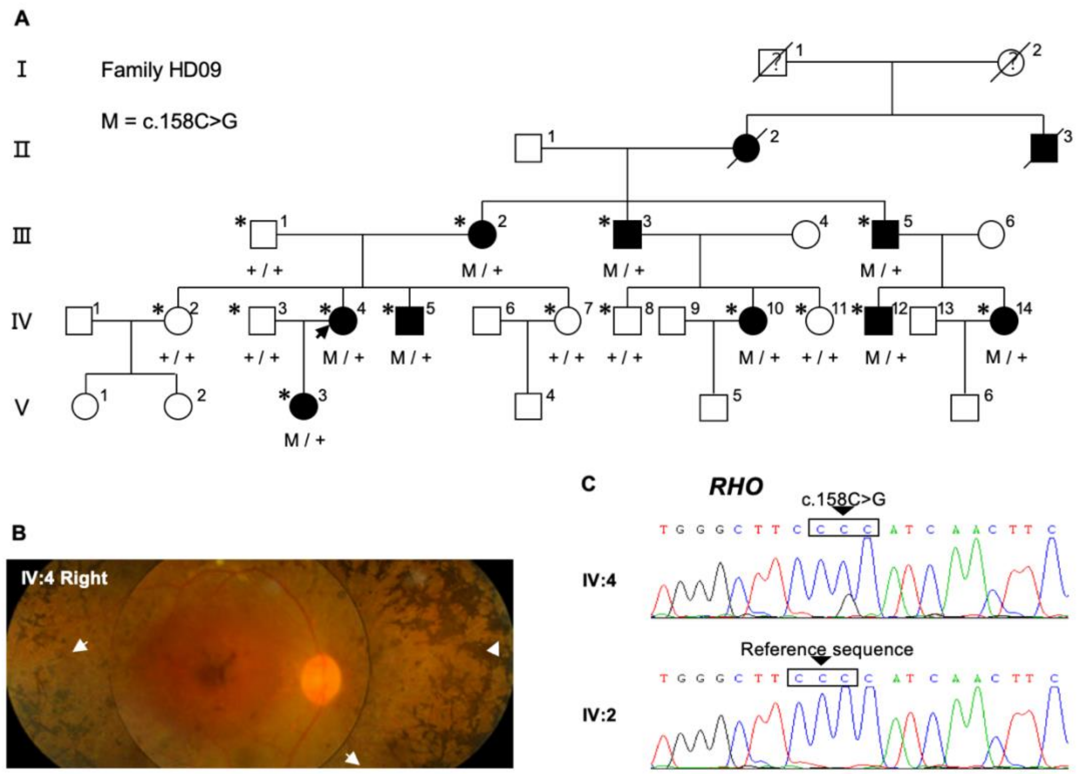

Supplemental Fig. 1. Pedigree, clinic, and genetic evaluations of the HD09 family.

A: Pedigree of family HD09. Solid symbols, affected individuals; open symbols, unaffected individuals; arrow, proband; slash, deceased persons; +, wild-type; M, the heterozygous mutation c.158C>G (p.P53R) in the *RHO* gene; *, the family members available for the present study; **B**: Fundus photograph of right eyes; **C**: Sanger sequencing showing heterozygous c.158C>G in IV:4 (patient) and IV:2 (unaffected) respectively. Abbreviation: Het., heterozygous.
