# Supplementary material for: Variants identified by next-generation sequencing cause endoplasmic reticulum stress in Rhodopsin-associated retinitis pigmentosa
Source: BMC Ophthalmol. 2021 Oct 19;21:371. doi: 10.1186/s12886-021-02110-2 (PMC8525045; doi:10.1186/s12886-021-02110-2)
Supplement: Supplementary file 2 — Additional file 2. [file 12886_2021_2110_MOESM2_ESM.pdf]

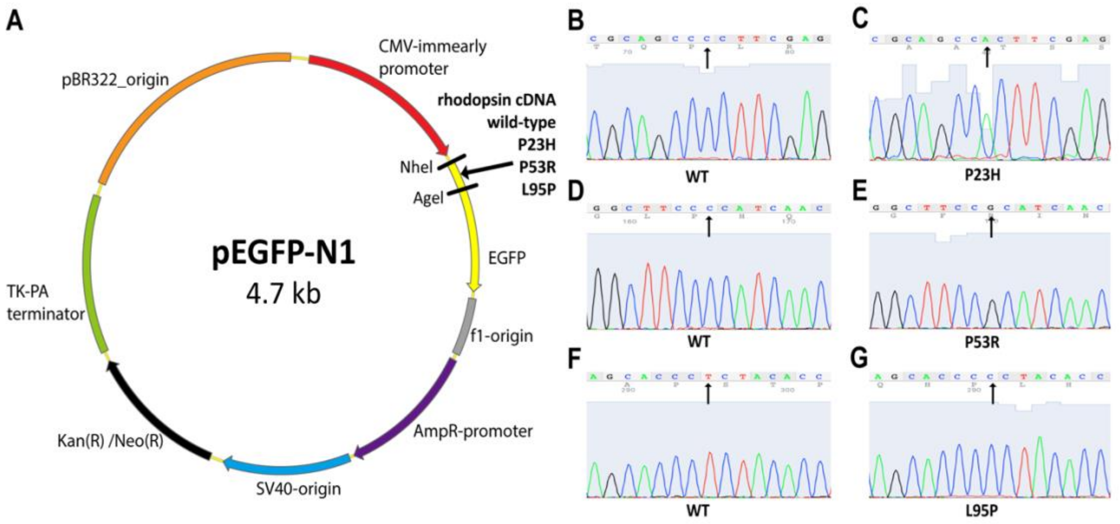

Supplemental Fig. 2. Plasmid construction and sequencing. A: The wild-type rhodopsin cDNA and p.P23H, p.P53R, p.L95P mutant rhodopsin cDNA were cloned into the pEGFP-N1 expression plasmid; **B-G**: Sanger sequencing of plasmids after construction.
