# Supplementary material for: Variants identified by next-generation sequencing cause endoplasmic reticulum stress in Rhodopsin-associated retinitis pigmentosa
Source: BMC Ophthalmol. 2021 Oct 19;21:371. doi: 10.1186/s12886-021-02110-2 (PMC8525045; doi:10.1186/s12886-021-02110-2)
Supplement: Supplementary file 3 — Additional file 3. [file 12886_2021_2110_MOESM3_ESM.pdf]

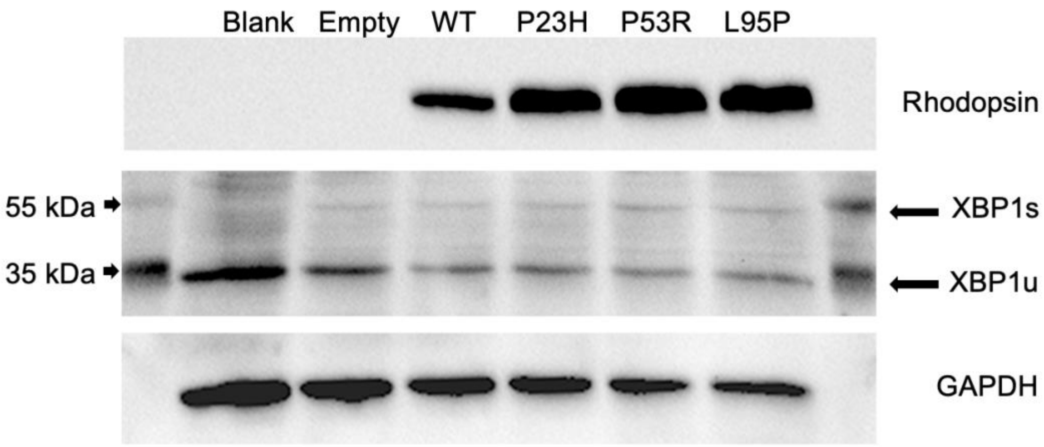

Supplemental Fig. 3. Detection of *XBP1* splicing. Splicing of *XBP1* in protein level was detected using western blot analysis. The IRE1-dependent ER stress pathway was activated in mRNA level, but is not clear in protein
