# Supplementary material for: Variants identified by next-generation sequencing cause endoplasmic reticulum stress in Rhodopsin-associated retinitis pigmentosa
Source: BMC Ophthalmol. 2021 Oct 19;21:371. doi: 10.1186/s12886-021-02110-2 (PMC8525045; doi:10.1186/s12886-021-02110-2)
Supplement: Supplementary file 4 — Additional file 4. [file 12886_2021_2110_MOESM4_ESM.pdf]

Supplementary Information file (Raw data)

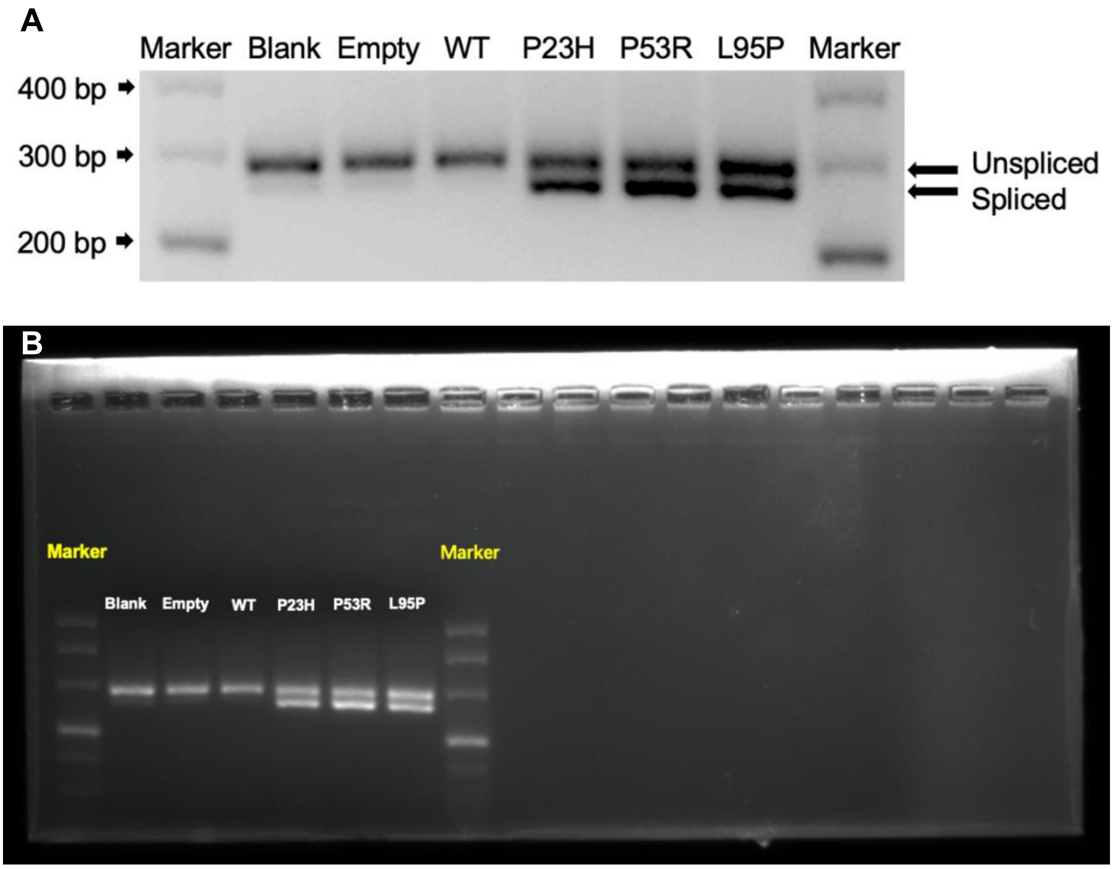

Original gel data to Fig.3 in manuscript. A: Fig.3 in manuscript. B: Original gel data to Fig.3.

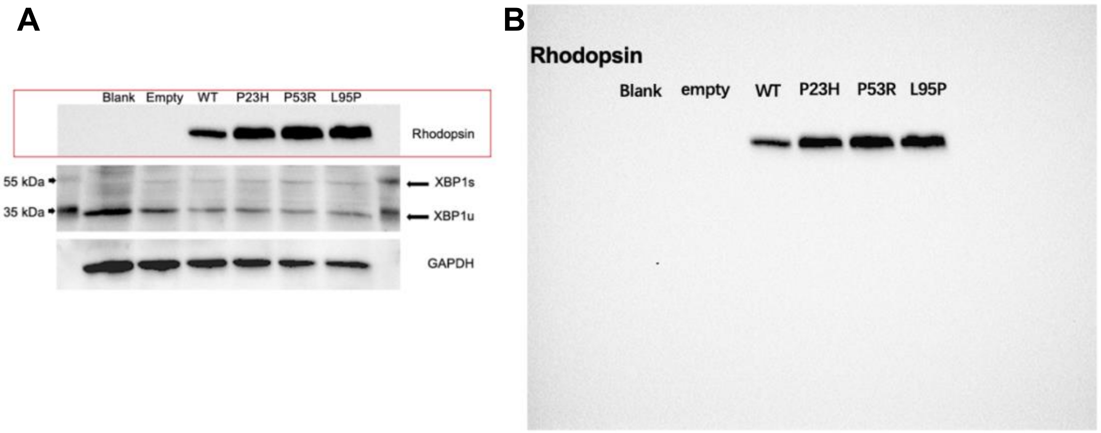

Original blot data to Supplementary Fig.3 in manuscript. A: Supplementary Fig.3 in manuscript. **B**: Original blot data to Rhodopsin band.

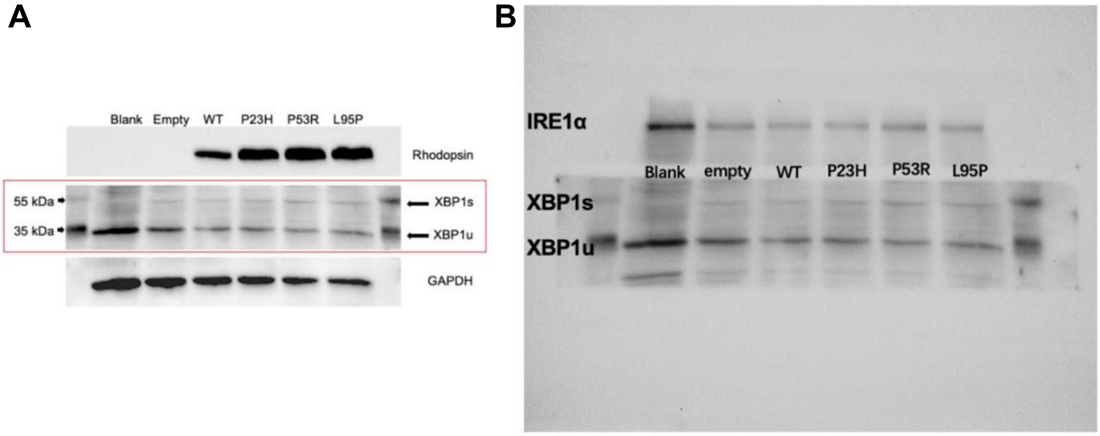

Original blot data to Supplementary Fig.3 in manuscript. A: Supplementary Fig.3 in manuscript. **B**: Original blot data to XBP1 band.

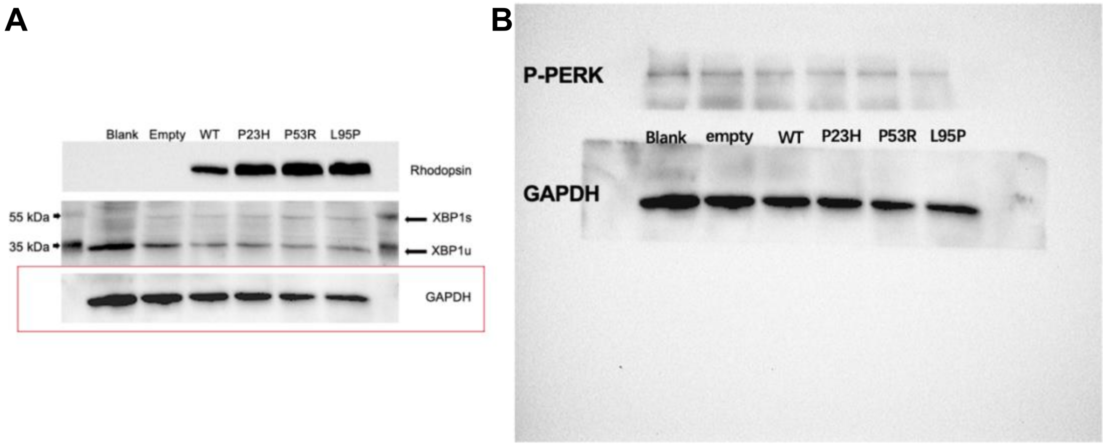

Original blot data to Supplementary Fig.3 in manuscript. A: Supplementary Fig.3 in manuscript. **B**: Original blot data to GAPDH band.
